# Transcriptome changes in age-related macular degeneration

**DOI:** 10.1186/1741-7015-10-21

**Published:** 2012-02-27

**Authors:** S Scott Whitmore, Robert F Mullins

**Affiliations:** 1 Department of Ophthalmology and Visual Sciences, The University of Iowa Carver College of Medicine, 4135E MERF, 375 Newton Rd., Iowa City, IA 52242, USA; 2 Center for Bioinformatics and Computational Biology, The University of Iowa, 5315 SC, 103 South Capitol St., Iowa City, IA 52242, USA

**Keywords:** age-related macular degeneration, transcriptome, systems biology

## Abstract

Age-related macular degeneration (AMD) is a debilitating, common cause of visual impairment. While the last decade has seen great progress in understanding the pathophysiology of AMD, the molecular changes that occur in eyes with AMD are still poorly understood. In the current issue of *Genome Medicine*, Newman and colleagues present the first systematic transcriptional profile analysis of AMD-affected tissues, providing a comprehensive set of expression data for different regions (macula versus periphery), tissues (retina versus retinal pigment epithelium (RPE)/choroid), and disease state (control versus early or advanced AMD). Their findings will serve as a foundation for additional systems-level research into the pathogenesis of this blinding disease.

Please see related article: http://genomemedicine.com/content/4/2/16

## Background

Age-related macular degeneration (AMD) is a progressive, complex disease, representing the most common cause of legal blindness in the developed world. In the United States alone, the prevalence of any type of AMD in individuals over the age of 40 is conservatively about 6.5%, or 7.2 million [1]. Risk factors for AMD include age, smoking, and a number of genetic polymorphisms.

**Figure 1:**
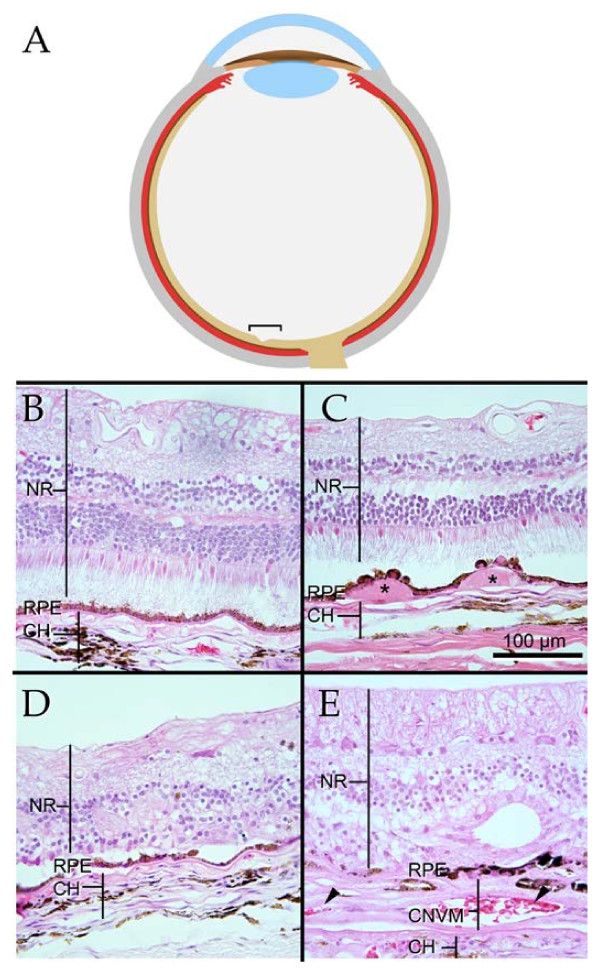
**Structural features of eyes with AMD**. (**A**) Schematic of human eye with the macular region indicated by the bracket. (B-E) Histological sections of eyes from individuals with normal retina (**B**); drusen (asterisks) beneath the RPE, a sign of early stage AMD (**C**); geographic atrophy with loss of photoreceptor cells (**D**); and choroidal neovascularization, with pathologic angiogenesis beneath the retina and RPE (**E**). The paper by Newman and colleagues explored gene expression differences in neural retina and the RPE/choroid layers based on disease state and region in an unprecedented number of eyes. asterisks, drusen; arrowheads in E, pathologic blood vessels. AMD, age-related macular degeneration; CH, choroid; CNVM, choroidal neovascular membrane; NR, neural retina; RPE, retinal pigment epithelium.

Early stages of AMD are clinically characterized by changes in retinal pigment epithelium (RPE) pigmentation and accumulation of drusen, extracellular deposits beneath the RPE. In some patients, AMD progresses to severe retinal atrophy and/or the pathologic growth of blood vessels from the choroid into the retina, a destructive process called choroidal neovascularization (CNV) (Figure 1). For reasons not completely understood, these events are predominantly localized to the macula, a specialized region of the retina that performs the crucial task of delivering sharp, central vision. Loss of macular photoreceptor cells results in inability to read, drive, or recognize faces. Several biological processes have been implicated in the pathogenesis of AMD, including complement-activation [2], inflammation [3], and oxidative stress [4].

While the last decade has seen great progress in understanding the pathophysiology of AMD, the molecular changes that occur in eyes with AMD are still poorly understood. The study of AMD is complicated by the limitations of animal models (for example, apart from primates, mammals lack a specialized macula), necessitating the use of human tissue. Anti-angiogenic drugs have been used to suppress CNV; however, no treatments are currently available to halt AMD prior to irreversible retinal damage, although dietary modulation may provide some benefit [5,6]. Identification of potential therapies may be facilitated by high-throughput systems biological analyses, particularly at the proteome and transcriptome levels.

Previous studies assessing mRNA levels in normal human retina, RPE, and choroid revealed tissue-specific molecular signatures [7,8] and differences between macular and extramacular transcript expression [9-13]. However, systematic transcriptional profiling had not been performed on AMD affected tissues, and the overall molecular phenotypes of AMD have not been thoroughly examined.

In the current issue of *Genome Medicine*, Newman and colleagues address this gap. The authors compared gene expression of 37 AMD eyes (divided into categories based on disease phenotype) with 31 normal eyes [14]. Expression profiling was performed for both the neural retina and the combined RPE/choroid layers of the eye using an oligonucleotide microarray platform. These experiments uncovered distinct molecular signatures for each assigned AMD class, termed disease modules, as well as a set of differentially regulated genes shared by all classes. By overlapping disease modules with existing protein association data, Newman *et al*. constructed interaction networks (interactomes) for AMD in RPE/choroid and retina.

## Discussion

### Identification of disease modules associated with AMD grade

Standard differential expression analysis produced only a small number of statistically significant genes associated with AMD classes. The authors then relaxed their filtering criteria and computationally identified modules of genes within and among AMD classes, tissue types, and anatomical regions (macula and periphery) based on coordinate expression and putative shared biological function. This elegant clustering method, designed to detect patterns confounded by background variation, revealed that the RPE/choroid in eyes with CNV showed increased expression of genes associated with angiogenic processes (such as VCAM1 and WNT4), whereas samples with geographic atrophy (GA) were enriched for apoptosis-related genes (such as caspases).

In both the RPE/choroid and retina, genes elevated across all AMD grades were enriched for regulators of cell-mediated immunity. In the RPE/choroid, these included immunoglobulin genes, a number of cytokines, and several CD antigens associated with T cell activation. These findings provide strong support for the concept that the microenvironment in AMD eyes is pro-inflammatory, with increased numbers and/or activities of leukocytes that may be responsible for injury to resident RPE or choroidal cells. Moreover, the elevation of these transcripts at the earliest stages of AMD, even before disease may be clinically identified, may suggest early opportunities for therapeutic management, which would be a major advance.

### Prediction of AMD status based on common molecular signature

One potentially important metric to validate the molecular signatures of the AMD eyes is to determine the predictive power of the disease modules to correctly assign new samples into the appropriate group. The authors constructed a computational classification model based on the twenty most significant genes from the globally upregulated RPE/choroid module. After training the model on subsets of the original cohort of microarrays, Newman *et al*. tested it on a second cohort of 47 microarrays. The model performed well, correctly classifying an average of 71% of the samples in the second cohort as either 'AMD' or 'normal. This result supports the finding of a globally upregulated set of genes across AMD stages in the RPE/choroid.

### Functional enrichment of genes within protein interactomes

A hallmark of many systems-level studies is the integration of large datasets with molecular networks. These models become frameworks for further analyses, such as the identification of pathologically altered pathways or the discovery of central modulators of network function, which may serve as drug targets. Newman *et al*. used genes from statistically significant disease modules to retrieve protein association relationships from online interaction repositories. From this information, they constructed interactomes for RPE/choroid and retina. Data regarding macular versus extramacular expression and disease progression was then overlaid on the network. Both interactomes were significantly enriched for genes previously associated with AMD. In the macular retina, phototransduction elements decreased expression with increasing AMD severity. This measured decrease likely results from the loss of rod and cone photoreceptor cells observed in advanced AMD (for example, Figure 1D, E).

### Areas for additional study

While the study by Newman *et al*. is notable as the first transcriptomics study to systematically analyze AMD cases and to so do with a very large sample cohort, several issues remain to be addressed by the field. Presumably, most of the mRNA changes detected will result in altered levels of their gene products. However, the relationship between changes in mRNA expression and protein production are not always linear. For example, a recent study analyzed gene expression in porcine tissues using three platforms (two that interrogated RNA levels and one that quantified proteins) [15]. By comparing the ratios of positively correlated genes common to the three technologies, the authors found good correspondence between transcript ratios and protein ratios for about 75% of these genes, with approximately 25% showing poor correspondence. Thus, integration of transcriptome profiling, as presented by Newman *et al*., with new and previously published proteomics surveys of AMD tissue [16-18] may resolve which transcripts are translated directly or are under post-transcriptional regulation.

In addition, methods are now available to assess not only the total abundance of mRNA molecules encoded by a gene, but also their specific transcripts and novel isoforms [19,20]. Distinct splicing isoforms of several genes are associated with both inherited and acquired diseases [21] and identifying the specific isoforms of AMD-associated molecules may reveal novel regulatory mechanisms.

There are several potential sources for transcriptional signals that vary between affected and unaffected tissues, including: altered expression of the gene within resident cells; degeneration of a cell type in disease that contributes a specific transcript in the healthy organ; and increased migration/proliferation of a cell type such that a new synthetic source for the mRNA has been introduced. The elevation of cellular immunity genes in all stages of AMD is very interesting, and may represent altered behavior of resident RPE/choroidal cells and/or the introduction of increased numbers of leukocytes in the choroid of eyes with AMD, as described previously with immunohistochemistry [22,23]. As Newman and colleagues point out, identifying the cellular source(s) of the AMD-associated transcripts will be important extensions of this work.

Finally, a clearer picture has emerged over the last several years of how variants in genes associated with the complement pathways influence risk of AMD [24]. An extension of the excellent studies by Newman and colleagues would be comparing the transcriptome of anatomically healthy eyes at genetically low- or high-risk for developing AMD. Identifying altered patterns of complement proteins has been explored in human tissues with known AMD-associated genotypes [25,26]. Systems based analysis of tissues with different genotypes may uncover the initial molecular events in eyes predisposed to disease, even before the genesis of any overt pathology.

## Conclusions

The study by Newman *et al*. is a major contribution to the community, providing a comprehensive set of data for region-based (macular versus extramacular), tissue-based (retina versus RPE/choroid), and disease-based (control versus early, atrophic, and neovascular AMD) expression. Their findings will serve as a foundation for additional systems-level research including integration with proteomics, assessment of alternatively spliced transcripts, identification of novel transcripts, and determination of cell-specific contributions to gene expression. Systems-level approaches such as these will aid in unraveling the pathogenesis of this complex, blinding disorder.

## List of abbreviations

AMD: age-related macular degeneration; CNV: choroidal neovascularization; GA: geographic atrophy; RPE: retinal pigment epithelium.

## Competing interests

RM receives funding from Alcon Research, Ltd. to study the molecular biology and pathology of age-related macular degeneration. SSW has no competing interests.

## Authors' contributions

All authors wrote the manuscript. All authors read and approved the final manuscript.

## Pre-publication history

The pre-publication history for this paper can be accessed here:

http://www.biomedcentral.com/1741-7015/10/21/prepub
